# Infected Recurrent Thyroglossal Duct Cyst: A Case Report

**DOI:** 10.5811/cpcem.2020.4.46863

**Published:** 2020-07-02

**Authors:** Jennifer Foti, Felipe Grimaldo

**Affiliations:** Naval Medical Center San Diego, Department of Emergency Medicine, San Diego, California

**Keywords:** Thyroglossal duct cyst

## Abstract

**Introduction:**

A thyroglossal duct cyst (TGDC) is a congenital malformation in the neck. Surgical management is often recommended due to risk of recurrent infections and rare possibility of malignancy.

**Case Report:**

Herein, we describe the case of an adult presenting with tender neck mass and fever. She had a history of previous surgical excision of her TGDC as a child. On evaluation she was found to have a recurrent TGDC complicated by acute infection via computed tomography imaging.

**Conclusion:**

In patients who have had previous surgical intervention to remove a TGDC, recurrence with infection should remain a diagnostic consideration.

## INTRODUCTION

Thyroglossal duct cysts (TGDC) are a common congenital malformation, typically presenting in the pediatric population. Predominant occurrence is in the first decade of life with physical exam findings of a midline mobile mass at the level of the hyoid.^1^ Most cases are managed with surgical excision in childhood due to possibility of malignancy. While various surgical techniques aimed at removal have been previously described, risk of recurrence still remains high with an average of 11% of individuals experiencing this complication.^2^ Exact risk factors that predispose an individual for recurrence or timing of recurrence are not yet clear.^3^ However, this common complication is important when considering the differential of a patient with acute onset neck mass in the emergency department (ED). Here, we describe a patient who presented to the ED with a tender neck mass who was found to have recurrence of TGDC complicated by acute infection.

## CASE REPORT

A 24-year-old previously healthy female presented to the ED with four days of fever, sore throat, neck swelling, and voice change. She had been previously evaluated at an urgent care facility where she had a negative rapid antigen detection test for group A streptococcus, but was referred to the ED for suspected peritonsillar abscess. Her past medical history was significant for tonsillectomy, adenoidectomy, and TGDC excision. On physical exam, she was appropriately managing her secretions, protecting her airway, and was able to lay supine without experiencing any respiratory distress. Her neck was diffusely swollen, with tender submandibular and anterior cervical lymphadenopathy. Her voice was notably muffled. No discrete fluctuant mass could be palpated or visibly appreciated. The remainder of her physical exam was unremarkable and her vital signs were within normal limits.

Computed tomography (CT) of the neck with contrast revealed a 1.0 × 1.3 × 2 centimeter midline lobulated fluid collection with mild rim enhancement immediately anterior to the lingual tonsils (Image). The otolaryngology service was consulted in the ED. Flexible fiberoptic laryngoscopy was performed at bedside, which revealed a patent airway with an edematous base of the tongue abutting the epiglottis and limited view of the vallecula. Given the history, exam, and radiographic findings, the diagnosis of recurrent, infected TGDC was made. The patient was started on intravenous ampicillin-sulbactam and admitted to the hospital. After resolution of the acute infection, the patient returned four weeks later for excision of the recurrent TGDC.

## DISCUSSION

The differential diagnosis for neck swelling, and voice change includes infectious etiologies, oncologic processes, lymphadenopathy, and various cysts. As previously mentioned, the majority of TGDCs will present in childhood, with 60% before age 20. While infrequent, primary occurrence can also present in adulthood, with an even distribution between males and females.^1^ A majority of initial presentations are asymptomatic, but a TGDC may be complicated by infection or fistula formation. Patients who experience these complications usually present with dysphagia, throat pain, or tender neck swelling. Infections of a first-time TGDC occur in approximately 8% of patients. Regardless of presentation, definitive management remains surgical excision.^3^

Recurrence rate following excision remains uncommon. The earlier in childhood the excision is performed, the higher the rate of recurrence.^3^ However, in the adult population it is important to include recurrence of TGDC as part of the differential diagnosis despite removal in childhood as demonstrated in this case. From our review of the literature, the rate of recurrence complicated by infection is unknown and exceedingly uncommon. This is the first case report that describes recurrence of a TGDC that is also complicated by infection. Diagnosis can be challenging, and ultimately requires imaging to further characterize. CT imaging will demonstrate a midline cystic mass with ring enhancement.^4^ In the acute infectious stage, patients are at risk for airway compromise due to significant swelling and should be admitted to the hospital for intravenous antibiotics, close observation and otolaryngology consultation. Definitive management for TGDC remains surgical excision once the infection has cleared.^1^

## CONCLUSION

Although infection of a recurrent TGDC is rare, early recognition and diagnosis in the ED is key to appropriate dispositioning of patients, and avoidance of potential airway compromise.

**CPC-EM Capsule**

What do we already know about this clinical entity?

Thyroglossal duct cysts (TGDC) commonly present in childhood as benign midline neck masses, usually treated with surgical resection.

What makes this presentation of disease reportable?

This is the first case report detailing a recurrent TGDC complicated by acute infection.

What is the major learning point?

Acute infected TGDC should remain on the differential diagnosis for neck swelling regardless of history of previous excision.

How might this improve emergency medicine practice?

As emergency clinicians it is important to keep common childhood pathologies and their complications on the differential even into adulthood.

## Figures and Tables

**Image:**
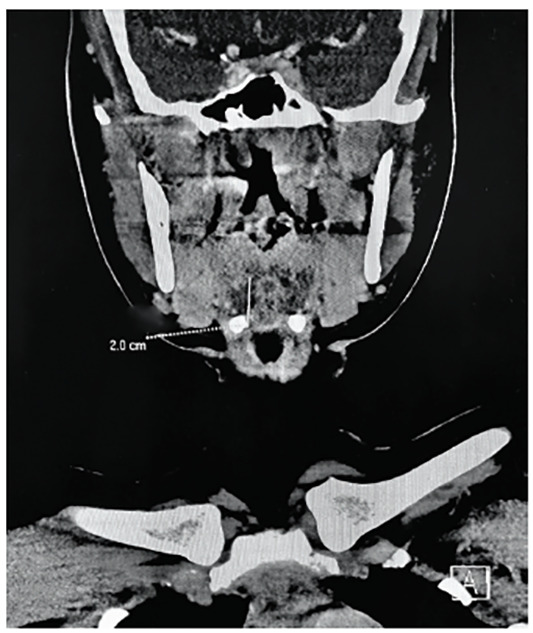
1.0 × 1.3 × 2 centimeter midline lobulated fluid collection in patient with infected, recurrent thyroglossal duct cyst.
